# Safety and Efficacy of Ipilimumab plus Nivolumab and Sequential Selective Internal Radiation Therapy in Hepatic and Extrahepatic Metastatic Uveal Melanoma

**DOI:** 10.3390/cancers14051162

**Published:** 2022-02-24

**Authors:** Veronica Aedo-Lopez, Camille L. Gérard, Sarah Boughdad, Bianca Gautron Moura, Gregoire Berthod, Antonia Digklia, Krisztian Homicsko, Niklaus Schaefer, Rafael Duran, Michel A. Cuendet, Olivier Michielin

**Affiliations:** 1 Department of Oncology, Lausanne University Hospital (CHUV), Rue du Bugnon 46, CH-1011 Lausanne, Switzerland; veronica.aedolopez@monashhealth.org (V.A.-L.); bianca.gautronmoura@h-fr.ch (B.G.M.); gregoire.berthod@chuv.ch (G.B.); antonia.digklia@chuv.ch (A.D.); krisztian.homicsko@chuv.ch (K.H.); 2 Department of Oncology, Monash Medical Centre, 823-865 Centre Road, East Bentleigh, Melbourne, VIC 3165, Australia; 3 Precision Oncology Center, Lausanne University Hospital (CHUV), Rue du Bugnon 46, CH-1011 Lausanne, Switzerland; camille.gerard@chuv.ch (C.L.G.); michel.cuendet@chuv.ch (M.A.C.); 4 Department of Nuclear Medicine and Molecular Imaging, Lausanne University Hospital (CHUV), Rue du Bugnon 46, CH-1011 Lausanne, Switzerland; sarah.boughdad@chuv.ch (S.B.); niklaus.schaefer@chuv.ch (N.S.); 5 Department of Radiology and Interventional Radiology, Lausanne University Hospital (CHUV), Rue du Bugnon 46, CH-1011 Lausanne, Switzerland; rafael.duran@chuv.ch; 6 Molecular Modelling Group, Swiss Institute of Bioinformatics, Quartier Sorge, CH-1015 Lausanne, Switzerland

**Keywords:** uveal melanoma, immune checkpoint inhibitors, selective internal radiation therapy

## Abstract

**Simple Summary:**

Despite recent progress on the treatment of metastatic uveal melanoma (mUM), prognosis remains dismal for the majority of patients. Directed liver therapies including selective internal radiation therapy (SIRT) have been the pillar of hepatic metastases management. Independently, immune checkpoint blockade by combination of ipilimumab plus nivolumab has demonstrated a median survival slightly superior to 1 year. However, the benefit of sequential ipilimumab plus nivolumab immunotherapy and SIRT has not been elucidated.

**Abstract:**

To assess the safety and efficacy of ipilimumab plus nivolumab around selective internal radiation therapy (SIRT) in patients with metastatic uveal melanoma (mUM). We present a retrospective, single center study of 32 patients with mUM divided into two groups based on the treatment received between April 2013 and April 2021. The SIRT_IpiNivo cohort was treated with Yttrium-90 microspheres and ipilimumab plus nivolumab before or after the SIRT (*n =* 18). The SIRT cohort underwent SIRT but did not receive combined immunotherapy with ipilimumab plus nivolumab (*n =* 14). Twelve patients (66.7%) of the SIRT_IpiNivo arm received SIRT as first-line treatment and six patients (33.3%) received ipilimumab plus nivolumab prior to SIRT. In the SIRT group, seven patients (50.0%) received single-agent immunotherapy. One patient treated with combined immunotherapy 68 months after the SIRT was included in this group. At the start of ipilimumab plus nivolumab, 94.4% (*n =* 17) presented hepatic metastases and 72.2% (*n =* 13) had extra liver disease. Eight patients (44.4%) of the SIRT_IpiNivo group experienced grade 3 or 4 immune related adverse events, mainly colitis and hepatitis. Median overall survival from the diagnosis of metastases was 49.6 months (95% confidence interval (CI); 24.1-not available (NA)) in the SIRT_IpiNivo group compared with 13.6 months (95% CI; 11.5-NA) in the SIRT group (log-rank *p*-value 0.027). The presence of extra liver metastases at the time of SIRT, largest liver lesion more than 8 cm (M1c) and liver tumor volume negatively impacted the survival. This real-world cohort suggests that a sequential treatment of ipilimumab plus nivolumab and SIRT is a well-tolerated therapeutic approach with promising survival rates.

## 1. Introduction

Uveal melanoma (UM) is a rare cancer with an incidence in Europe that varies from <2 per million in Spain and southern Italy up to >8 per million in Northern countries such as Norway or Denmark [1]. However, it is the most frequent primary intraocular malignancy. Despite effective therapy for primary tumors, 50% of patients will develop metastases, principally in the liver [2,3,4]. The prognosis of metastatic disease is poor with survival rates around 20% at 1 year, and 10% at 2 years [3,4,5]. Long-term survival after diagnosis of metastasis is uncommon.

Different systemic treatments have been studied including chemotherapy, targeted therapy and immunotherapy but standard of care does not exist for metastatic uveal melanoma (mUM) patients yet. Most chemotherapies presented overall response rates (ORR) under 5% [6]. Single-agent chemotherapy such as fotemustine, dacarbazine, temozolomide and cisplatin, as well as combined regimens, such as dacarbazine–treosulfan or gemcitabine–treosulfan was investigated with disappointing results [6,7,8,9,10]. Regarding targeted therapy, although selumetinib demonstrated activity in a randomized phase 2 trial, selumetinib associated to dacarbazine did not improve progression-free survival (PFS) compared to dacarbazine and placebo in the phase 3 study [11,12]. Additionally, inhibitors targeting the PI3K/AKT/MTOR pathway showed no benefit or demonstrated limited clinical benefit in phase 2 trials [13,14,15]. Recently, a protein kinase C inhibitor showed modest clinical activity in a phase I study [16]. Lately, tebentafusp, a bispecific antibody that redirects T cell lysis of melanoma cells expressing gp100, demonstrated a prolonged overall survival (OS) as first-line therapy for mUM patients with HLA-A*02:01 compared to investigator’s choice (IC), either pembrolizumab, ipilimumab or dacarbazine [17].

Although immune checkpoint inhibitors revolutionized the prognosis of cutaneous melanoma, similar outcomes were not reached in mUM. Immunotherapy based on the single CTLA4 and PD1 checkpoint blockade demonstrated limited activity in mUM [18,19]. PFS of patients treated with ipilimumab varied between 2.8 and 3.6 months and OS between 6.8 and 9.6 months in different trials [20,21]. Anti-PD1 and anti-PDL1 therapies such as pembrolizumab, nivolumab or atezolizumab alone also showed disappointing results with ORR of 3.6% and median overall survival (mOS) of 7.6 months [22]. However, the combination of ipilimumab and nivolumab resulted in longer OS than single-agent immunotherapy, surpassing 1 year in different cohorts with ORR that varies from 11.5 up to 18% [23,24,25,26,27].

Given that the liver is the most common site of metastasis affecting up to 90% of patients with mUM, local strategies to treat hepatic disease were widely studied [28,29]. Surgery improved OS in cases of complete resection, nevertheless, only a limited number of patients are eligible for surgical intervention due to the presence of multiple lesions or multi lobar involvement [30]. Liver-directed therapies such as radiofrequency ablation, radiotherapy, chemoembolization, immunoembolization, radioembolization, isolated hepatic perfusion and percutaneous hepatic perfusion, was employed to treat metastatic liver disease [10,31]. Selective internal radiation therapy (SIRT) was demonstrated to be effective in mUM patients with less than 25% of tumor burden [32], as salvage therapy [33] and as first-line treatment [34].

Studies suggested that radiotherapy and immunotherapy synergize to enhance the efficacy of the treatments [35]. Two case-series described clinical outcomes of 11 and 12 mUM patients treated by SIRT and sequential immune checkpoints inhibitors. Most of the patients in these studies received either CTLA4 or PD1 inhibitors in monotherapy and the mOS described was around 1.5 years [36,37]. Furthermore, a retrospective review showed a larger mOS with SIRT and concurrent single-agent immunotherapy by ipilimumab, pembrolizumab, nivolumab or IL2 compared to SIRT alone (26 versus 9.5 months) [38]. Few data are currently available on the benefits of combined immunotherapy of ipilimumab plus nivolumab before or after a treatment by SIRT. The objective of our study is to analyze the safety and efficacy of SIRT and sequential immunotherapy combination versus SIRT without combined immunotherapy in patients with mUM.

## 2. Materials and Methods

### 2.1. Patients

We conducted a single center, observational and retrospective study of mUM patients treated by SIRT between April 2013 and April 2021 at Lausanne University Hospital (CHUV), Switzerland. The population included 18 years and older patients with mUM histologically proven by liver biopsy who underwent SIRT treatment(s) in our center. The analysis was conducted in accordance with the Declaration of Helsinki, the Swiss legal requirements and the principles of good clinical practice. Patients signed the Lausanne University Hospital general consent and accepted the use of their data for research purposes or did not explicitly refuse the use of personal data (following Art. 34 HRA). The protocol was approved by the Research Ethics Committee of Canton de Vaud, Switzerland (protocol no. 2019-00448). Authorized qualified personnel of the CHUV Oncology Department retrieved personal and clinical data from electronic patient records. Available imaging was reviewed by a radiologist and a nuclear medicine radiologist. Date of death was obtained from the Swiss Federal Registry for the Persons. 

Patients were divided in two arms based on the treatment received. The SIRT_IpiNivo group included patients treated with Yttrium-90 microspheres and ipilimumab plus nivolumab before or after the SIRT. The median time between the first SIRT and the start of ipilimumab plus nivolumab was 7.7 months (95% CI; 7.3–13.0 months). The SIRT group included patients who underwent a SIRT but did not receive ipilimumab plus nivolumab. However, in the SIRT group, 8 patients (57.1%) received immunotherapy. Five of these 8 patients started anti-CTLA4 alone after SIRT and 2 received single anti-PD1, 1 before SIRT and 1 after SIRT. One patient treated with combined immunotherapy 68 months after the SIRT was included in this group due to the very long delay between SIRT and the immunotherapy treatment. A total of 32 patients were included in this study, 18 in the SIRT_IpiNivo group and 14 in the SIRT group. 

Six patients (33.3%) of the SIRT_IpiNivo group received ipilimumab plus nivolumab treatment prior to the SIRT and the SIRT was performed as first-line therapy in the remaining 12 patients (66.7%). All patients presented a progressive disease (PD) before they received the next treatment, either SIRT or ipilimumab plus nivolumab.

### 2.2. Treatments

The systemic therapy consisted of ipilimumab 3 mg/kg combined with nivolumab 1 mg/kg every 3 weeks for a total of 4 doses followed by nivolumab 3 mg/kg or 240 mg flat dose every 2 weeks. In case of grade 3–4 toxicity or progression, the therapy was interrupted.

The ^90^Y microspheres procedure was carried out according to previously published guidelines [39,40]. Patients underwent a simulation angiography to embolize non-target extrahepatic vessels and avoid any unintentional transmission of the microspheres to non-selected organs. Then, Technetium-99m macro aggregated albumin (99mTc-MAA) was injected into the selected hepatic artery to further assess lung or digestive shunting prior to therapy, tumoral volume targeting and dosimetry. Weeks later, the SIRT was performed as planned to one or both lobes. Whole liver could also be treated in more than one session. 

### 2.3. Response Evaluation and Toxicity Analysis

Patients underwent imaging (whole body Positron Emission Tomography (PET) scan, liver Magnetic Resonance Imaging (MRI) and/or contrast-enhanced Computed Tomography (CT)) before starting the treatment, and then every 3 months. Liver tumor response to the SIRT was assessed by the modified Response Evaluation Criteria in Solid Tumors (mRECIST) and response to the immunotherapy was evaluated by PET Response Criteria In Solid Tumors (PERCIST) version 1.1. To evaluate liver response to the SIRT, target lesions were assessed at 3 and 6 months after the end of treatment. Adverse events related to the treatments were classified following the Common Terminology Criteria of Adverse Events (CTCAE) version 4.0. Complications related to the SIRT were collected until 30 days after the procedure.

### 2.4. Statistical Analysis

OS was analyzed from the first treatment, either radioembolization or ipilimumab plus nivolumab, from diagnosis of metastases and from the first SIRT performed until death or last follow-up. Hepatic progression-free survival (hPFS) was calculated from the first SIRT administered to the liver progression after termination of SIRT treatments. To evaluate liver response to SIRT, the last radiological exam performed a maximum of 30 days before SIRT was considered and for ORR to immunotherapy the last radiological exam before combined immunotherapy. PFS post-SIRT was calculated from the first SIRT performed, and PFS post-immunotherapy from the first cycle of ipilimumab plus nivolumab until disease progression. Survival curves were calculated using the Kaplan–Meier method and the hazard ratio (HR) and 95% of CI using a Cox model. Within the R Statistical Computing environment v4.0.3, the packages used are survival, survminer and ggplot2 [41,42]. Significance is defined as a *p*-value < 0.05 for the log-rank test.

## 3. Results

### 3.1. Patients

Median age at diagnosis of metastases was 61 years in both groups. The median time from primary tumor diagnosis to the development of metastases was 28.8 and 21 months, respectively. Patient and SIRT treatment characteristics are summarized in Table 1.

At the time of SIRT, 8 patients (44.4%) in the SIRT_IpiNivo group and 3 patients (21.4%) in the SIRT group presented extra liver metastases. At the start of the immunotherapy combination, 94.4% (*n =* 17) of individuals of the SIRT_IpiNivo group presented with hepatic disease and 13 patients (72.2%) had extra liver metastases. Eleven patients (61.1%) presented with metastases in 3 or more organs. The most frequent site of extra liver metastases was lung (10 patients, 55.6%). Localization of extrahepatic metastases of the SIRT_IpiNivo group are described in Table 2.

Genomic testing was available in 88.9% of cases (*n =* 16) in the SIRT_IpiNivo group but only in 4 (28.6%) in the SIRT group. Hotspot mutations GNAQ or GNA11 were present in more than 90% of cases. In addition, somatic BAP1 mutation was found in 3 tumors (18.8%) and 1 tumor (6.2%) had a SF3B1 mutation. FGFR1 deletion and FGFR4 mutation were found in 2 different patients (11.1%). In the SIRT group, one tumor of a patient presented with SF3B1 mutation besides GNAQ mutation. Genomic characteristics are shown in Table 1.

### 3.2. Treatment Data

A total of 52 SIRT treatments were performed, 31 in the group SIRT_IpiNivo and 21 in the group SIRT. The median time from diagnosis of liver metastases to the first SIRT was 3.5 months (range: 1.0–29.8 months) in the SIRT_IpiNivo group and 2.3 months (range: 1.2–11.1 months) in the SIRT group. Median activity infused per patient was 2.4 GBq (range: 1.1–9 GBq) and 2.3 GBq (range: 1.3–5.4 GBq), respectively. SIRT treatment characteristics are shown in Table 1.

Concerning the ipilimumab plus nivolumab immunotherapy received by patients in the SIRT_IpiNivo group, it was the first systemic treatment for 16 of 18 patients (88.9%). The 2 remaining patients received sorafenib prior the immunotherapy as part of study protocol SIRT-Sorafenib (NCT01893099). Eleven patients (61.1%) completed 4 cycles of ipilimumab plus nivolumab. Three of these patients did not continue maintenance therapy with nivolumab due to PD. Characteristics of ipilimumab plus nivolumab treatment received by patients of the SIRT_IpiNivo group are described in Table 2.

### 3.3. Toxicity

There were no deaths related to treatment. Complications related to SIRT and to ipilimumab plus nivolumab are summarized in Table 3. Immune-related adverse events (irAEs) were described in 12 patients (66.7%) of the SIRT_IpiNivo group. The irAEs most commonly developed were hepatitis and colitis. Eight patients (44.4%) presented with grade 3–4 complications, all of which received corticoids. Four cases needed a second immunosuppressive drug. Three of 7 patients who discontinued ipilimumab plus nivolumab due to toxicity, resumed immunotherapy with single-agent checkpoint nivolumab, as soon as the immune related complication was resolved. These patients discontinued nivolumab later, 2 cases due to immune related complications and the remaining case due to PD.

Regarding toxicities related to the SIRT, a grade 3 complication was described in 1 patient of each group. The most frequent complication was abdominal pain. The grade 3 complications were a celiac arterial dissection treated by angioplasty and an enterocolitis managed by intravenous antibiotics. Importantly, 10 patients (55.6%) of the SIRT_IpiNivo group and 6 patients (42.8%) of the SIRT group presented with abnormal hepatic tests before the SIRT was performed. 

### 3.4. Outcomes

**Figure 1:**
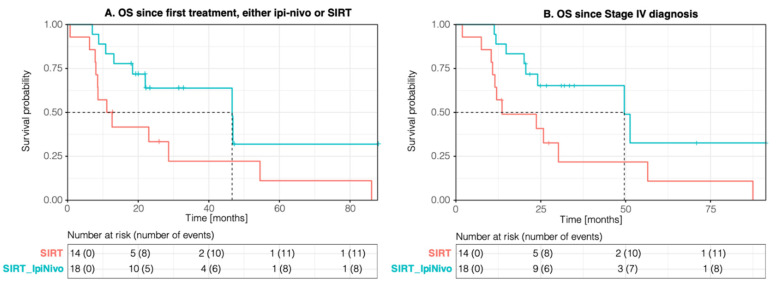

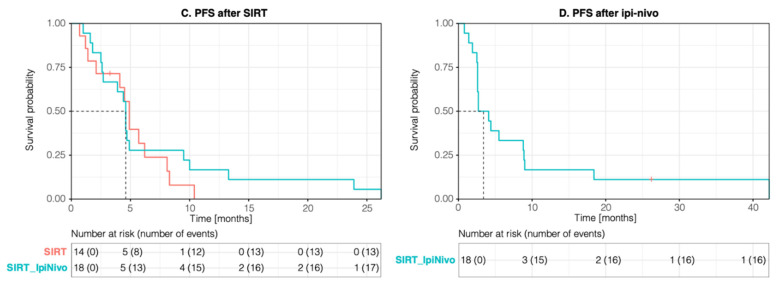
Kaplan–Meier curves for overall survival (OS) since first treatment (**A**) and from the diagnosis of metastases (**B**). Kaplan–Meier curve for progression-free survival (PFS) from SIRT (**C**) and from ipilimumab plus nivolumab (ipi-nivo) (**D**).

The median follow-up from the diagnosis of metastases was 23.9 months (range: 1.9–91.3 months). The mOS from the diagnosis of metastatic disease was 49.6 (95% CI; 24.1-NA months) in the SIRT_IpiNivo group compared to 13.6 (95% CI; 11.5-NA) in the SIRT group (*p*-value 0.027), while mOS from the first treatment was 46.6 (95% CI; 22-NA) versus 11.8 (95% CI; 8.5-NA) months (*p*-value 0.039). The mOS from the first SIRT performed was 46.6 months (95% CI; 18.4-NA) and 11.1 months (95% CI; 8.0-NA), respectively (*p*-value 0.1) (Figure 1). There was no statistically significant difference in mOS between patients of the SIRT_IpiNivo group who underwent SIRT prior to or after immunotherapy. While the mOS was not reached for SIRT as first line, it was 46.6 months for ipilimumab plus nivolumab prior SIRT. 

**Figure 2:**
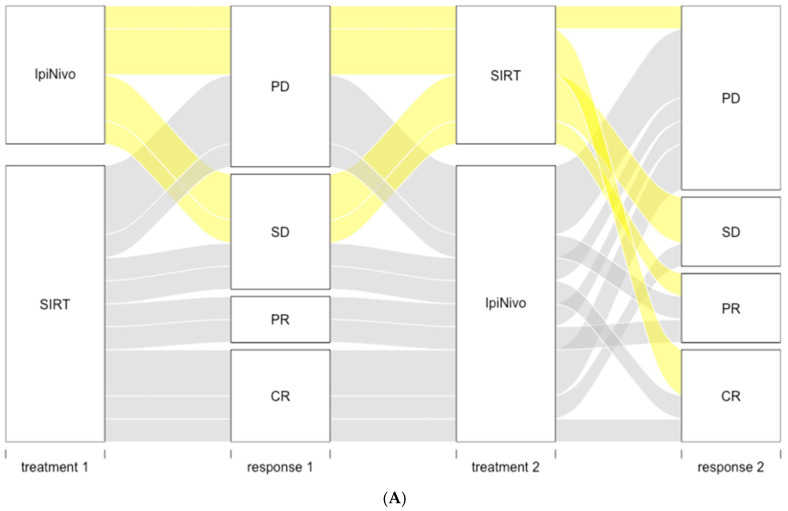

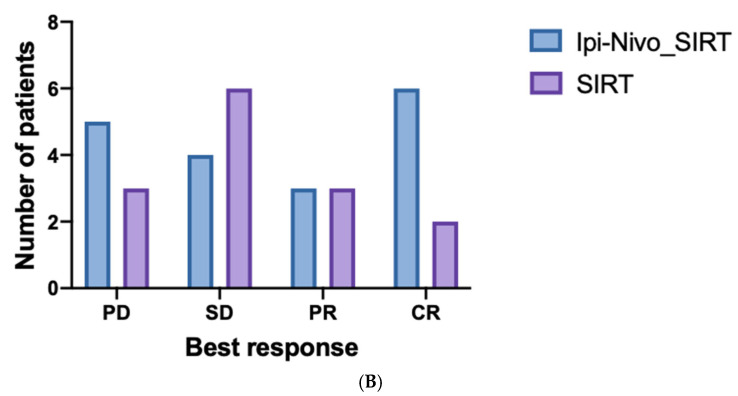
(**A**) Alluvial plot of best response to first treatment received, either SIRT or ipilimumab plus nivolumab (IpiNivo), and best response to the second treatment. (**B**) Liver responses to SIRT by group. CR: complete response; PR: partial response; SD: stable disease; PD: progressive disease.

Liver response following SIRT was observed in 9 (50%) and 5 (35.7%) patients of SIRT_IpiNivo and SIRT groups, respectively (Figure 2). Liver response to SIRT was comparable in the SIRT_IpiNivo group, regardless of whether ipilimumab plus nivolumab was administered prior to or after SIRT.

**Figure 3:**
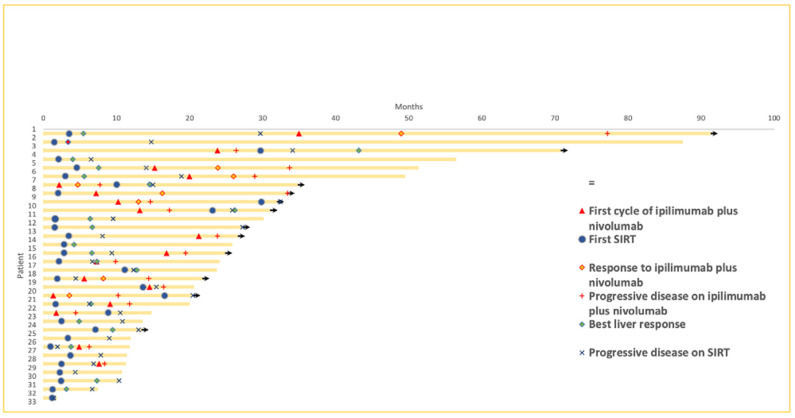
Swimmer plot. Follow up of each patient from the diagnosis of metastatic disease until death or end of study, time of start of ipilimumab plus nivolumab and first SIRT, best overall response to ipilimumab plus nivolumab and best liver response to SIRT and moment of progressive disease on ipilimumab plus nivolumab and on SIRT.

Median hPFS and median progression-free survival (mPFS) from the first SIRT was 8.7 (95% CI; 4.4-NA) and 4.6 (95% CI; 2.7–10) months in the SIRT_IpiNivo group and 5.6 (95% CI; 4.9-NA) and 4.9 (95% CI; 4.1-NA) months in the SIRT group (Figure 3). The median hepatic duration of response was 13.3 months (range: 2.1–28.5 months) in the SIRT_IpiNivo group versus 7.9 months (range: 4.9–15.3 months) in the SIRT group. Median hPFS was 8.7 months for patients who received SIRT prior to ipilimumab plus nivolumab and 10.2 months for upfront immunotherapy (no statistically significant difference).

At the time of PD following SIRT, 6 patients (33.3%) of the SIRT_IpiNivo group and 8 patients (57.1%) of the SIRT group presented progression of liver and extra liver metastasis; 3 and 2 patients, respectively (16.7% and 14.3%), had progression exclusively in the liver while 9 patients (50.0%) of the SIRT_IpiNivo group and 3 patients (21.4%) of the SIRT group presented only extra liver progression. Responses to SIRT at 3 and 6 months are summarized in Table S1. 

In the group SIRT_IpiNivo, ORR following ipilimumab plus nivolumab was 22.2% (*n =* 4). The disease control rate was 38.9% (*n =* 7). Two patients (11.1%) presented a complete response (CR). One of these two patients presented liver metastases only and the second patient presented with extrahepatic and hepatic metastases. This patient also had 3 millimetric brain metastases controlled by stereotaxic radiotherapy. The median duration of response (DoR) to immunotherapy for the SIRT_IpiNivo group was 22.3 months (range: 8.8–42.2 months). 

Univariate analysis was performed to identify factors influencing hPFS and OS (Table S2). Patients with an index liver tumor greater than 8 cm (M1c) at the first SIRT had decreased hPFS and OS from SIRT when compared with patients with the largest hepatic lesion smaller than 8 cm (*p*-value 0.00096; HR: 3.47 (1.19–10.07) for hPFS and *p*-value 0.00059; HR: 6.75 (1.95–23.35) for OS) (see Figure S1). Tumoral volume superior to the median (185 cc) also had a negative impact on hPFS (*p*-value 0.016; HR: 2.62 (1.17–5.86)) and OS from SIRT (*p*-value 0.046; HR: 2.82 (0.98–8.17)). Presence of extra liver metastatic disease at SIRT was prognostic but not predictive (*p*-value 0.034; HR: 2.82 (1.04–7.66) for OS from SIRT).

## 4. Discussion

This study describes the safety and efficacy of sequential combination of ipilimumab plus nivolumab and SIRT in patients with mUM compared with SIRT without combined immunotherapy. Our study showed that both therapies are well tolerated. Ipilimumab plus nivolumab before or after the SIRT was associated with improved OS in our retrospective analysis.

We analyzed complications related to SIRT as well as irAEs after immunotherapy combination. The frequency of grade 3 toxicities due to SIRT in our study was comparable to those described in other analyses [36,38]. Nevertheless, liver function test elevation was observed in more than 90% of patients in our study, which is notably higher than rates detailed previously, probably because of pre-existing liver test abnormalities presented by around half of our patients (55.5%). Most complications related to SIRT were treated conservatively. 

The safety profile of combined checkpoint inhibition of our analysis was also consistent with other cohorts. In our study, 44.4% of the SIRT_IpiNivo group developed grade 3 irAEs, whereas the prospective clinical trials of Piulats et al. and Pelster et al. who analyzed 52 and 35 patients with mUM treated by ipilimumab plus nivolumab, described grade 3 immune side effects in 57.7% and 40% of patients, respectively [26,27]. Najjar et al. described grade 3 immune side effects in 30% of an 89 mUM patient cohort treated with ipilimumab plus nivolumab [25]. The frequency of severe histologically proven grade 3 or 4 hepatitis was higher in our study (*n =* 4, 22.2%) compared with cutaneous melanoma patients treated with the same regimen. In the study of Larkin et al., 313 patients were treated with ipilimumab plus nivolumab and only 8.3% developed a severe increase in alanine amino-transferase and 6.1% of aspartate amino-transferase levels [43]. It is important to mention that the majority of patients in our study presented liver disease (94.4%) and pre-existing liver function test elevation (72%) at the start of immunotherapy and these conditions could eventually contribute to the hepatotoxicity developed by our patients. The frequency of immune-related hepatitis was greater with ipilimumab and nivolumab after SIRT (33.3%) than with upfront immunotherapy (16.7%).

As the sequence of treatments explored in our study was not homogenous, for instance a third of patients of the SIRT_IpiNivo group received immunotherapy prior SIRT and the remaining two thirds underwent SIRT before immunotherapy, to evaluate the benefit from the immunotherapy as well as the SIRT, we calculated mOS from the diagnosis of metastases and from the administration of the first treatment of either combined immunotherapy or SIRT. In both cases, mOS was significantly superior in the SIRT_IpiNivo group compared to the SIRT group. In addition, half of the SIRT group had previously received ipilimumab. However, as ORR to ipilimumab described in previous studies was extremely low, we did not expect a significant impact on the outcomes of this study. 

Additionally, a significant negative impact on survival rates from SIRT in univariate analysis were observed with the presence of extra liver metastases at the time of SIRT, larger liver lesions (M1c) and higher tumor volume (Table S2). The variables that reflect liver tumor burden also affected negatively the hPFS in our analysis, suggesting that a SIRT treatment should not be delayed in the presence of unresectable liver metastases. Patients with index liver metastasis greater than 8.0 cm (M1c according to AJCC 8th edition staging system) had negative repercussions in hPFS and OS from SIRT compared to those with smaller lesions. Levey et al. already described this effect when the largest liver lesion was greater than 7 cm [38].

Sequential treatment of SIRT and immunotherapy has been previously analyzed in retrospective studies [35,36,37]. Given that UM is a rare disease, these reports are limited by small sample size as it is the case of our study. Levey et al. analyzed 24 mUM patients treated by SIRT and concluded that the subgroup of 12 patients treated consecutively by SIRT and immunotherapy (ipilimumab, nivolumab, pembrolizumab or IL2) within 3 months before or after the SIRT, presented longer survival rates with regard to SIRT alone [38]. Ten patients of this review received the immunotherapy treatment prior to the SIRT. Ruohoniemi et al. presented a cohort of 22 patients, which included 12 mUM patients treated with radioembolization and immunotherapy with ipilimumab, nivolumab, pembrolizumab or ipilimumab plus nivolumab combination (*n =* 7) within a 15-month period [36] while Zheng et al. studied 11 patients treated by SIRT and anti-PD1 or anti-CTL4 alone. Nine of these cases received the immunotherapy before SIRT [37]. The mOS from the SIRT of these three studies was 18.6, 20 and 17 months, respectively. Survival rate from the diagnosis of liver disease of patients treated by SIRT and immunotherapy was reported by Zheng et al. and Levey et al. as 35.5 and 26 months, respectively. Additionally, Blomen et al. recently showed in a retrospective study of two cohorts, immunotherapy and liver directed therapy compared to standard therapies, that mOS showed significant improvement in the first cohort (22.5 versus 11 months) [44]. In our study, the mOS of the group treated by ipilimumab plus nivolumab and SIRT was 46.6 months (95% CI; 18.4-NA) from the SIRT and 49.6 (95% CI; 24.1-NA) from the diagnosis of metastases, compared with 11.1 (95% CI; 8.0-NA) and 13.6 (95% CI; 11.5-NA) months for those treated with SIRT without combined immunotherapy. Although a significant statistical difference was noted when the analysis was calculated from the diagnosis of metastasis (*p*-value = 0.027), the OS benefit with ipilimumab plus nivolumab was not statistically significant when survival was estimated from the SIRT. Whereas Levey et al. included only patients who received immunotherapy within 3 months of undergoing the SIRT, in our study six patients (33.3%) started ipilimumab plus nivolumab between 5.9 and 19.6 months before SIRT. Moreover, we cannot exclude a lead-time bias that would tend to overestimate the OS of the SIRT_IpiNivo group, due to the time interval between the treatments, during which no death can occur by definition. A comparative table recapitulating previous studies can be found in the Supplementary Materials (Table S3).

Although single-agent immunotherapy has not been effective in mUM, survival rates have been improved with combined CTLA4 and PD1 antibodies. In the retrospective analysis of Heppt et al. the mOS was 14 months for pembrolizumab, 10 months for nivolumab and it was not reached with combined immunotherapy. Nevertheless, the follow up for this group was only 3.9 months [23]. The largest cohort assessing combined immunotherapy in mUM patients, collected clinical data retrospectively from 89 patients [25]. Despite a modest PFS of 2.7 months, the mOS from treatment initiation was 15 months. Only two prospective studies have reported outcomes of combined ipilimumab plus nivolumab in mUM patients. GEM1402 included 50 treatment-naïve patients and PROSPER enrolled 35 patients accepting previously treated patients. In GEM1402, the mOS surpassed 1 year (12.7 months) despite mPFS being comparable to monotherapy [27]. PROSPER described 18% of ORR, 5.5 months of mPFS and 19.1 months of mOS. ORR and PFS since the 1st cycle of ipilimumab plus nivolumab of our analysis did not differ from those mentioned. While the ORR was 22.2%, mPFS was 4.4 (95% CI; 2.6–6.5) months [26]. However, comparison with previous trials should be done carefully as clinical characteristics differ largely between studies and differences may not be significant with this sample size. For instance, 50.0% of SIRT_IpiNivo patients had a high level of LDH versus 37.2% in GEM1402 and 43% in PROSPER. Furthermore, 8 of 9 patients (88.9%) of the SIRT_IpiNivo arm with BAP1 status available, exhibited a loss of expression of BAP1 associated with an increased risk of metastasis and a poor prognosis according to Robertson et al. [45]. SF3B1 mutated tumors may respond better to checkpoint inhibitors because SF3B1 alternative splicing may generate neo-antigens [46,47,48]. However, SF3B1 mutation is found in only 20%–25% of primary UM and this group has been classified as an intermediate metastatic risk [45]. Therefore, the frequency of SF3B1 mutation in our cohort is expected to be negligible. Regarding the distribution of metastases, whereas 67% of our patients presented with liver and extra liver disease at the first cycle of immunotherapy, 95% had liver involvement. GEM1402 and PROSPER reported hepatic and extrahepatic disease in 37% and 49% of patients, respectively. Moreover, around 20% of patients included in GEM1402 and PROSPER had extra liver metastases exclusively. Patients of GEM1402 with extra liver metastasis had longer survival rates regardless of liver status.

Regarding the response to SIRT, a systematic meta-analysis of 55 studies including 2446 patients evaluated different treatments of UM liver metastases and suggested an improvement of survival with surgery and locoregional procedures [29]. The mOS from the SIRT of five clinical studies reported in this meta-analysis varied between 2.9 and 12.3 months [32,33,49,50,51]. The largest cohort of mUM patients treated by SIRT was described by Eldredge-Hindy et al. and included 71 patients [33]. SIRT was administered as a salvage therapy in 82% of cases and the mOS was 12.3 months. When SIRT is administered as first line treatment, the mOS increases to 18 months as reported by Ponti et al. [34]. Tulokas et al. compared patients treated by SIRT to an historical mUM group without extrahepatic spread treated by systemic chemotherapy as first line treatment. The mOS was 13.5 months for patients treated by SIRT, significantly longer than the 10.5 months (*p*-value 0.047) of the historical group [52]; however, the mOS increased to 18.7 months with SIRT as first line treatment (*p*-value 0.017). In our cohort, 12 patients of every group received SIRT as the first line. The mOS since the first SIRT estimated for these patients, regardless of the group, was 22 months (95% CI; 10.8–86.1 months), comparable to studies previously described. Nevertheless, the mOS of patients managed with SIRT as the first line was 9.8 months (95% CI; 8.0-NA) for the SIRT group compared to 46.6 months (95% CI; 18.4-NA) in the SIRT_IpiNivo group. Despite a notably longer survival of patients treated with combined immunotherapy in addition to the SIRT, there was no statistically significant difference (*p*-value = 0.16) between both groups, but the moderate sample size of our cohort could have affected these results. We also reported a mPFS after SIRT comparable to the prospective phase 2 trial of Gonsalves et al. of 48 patients treated by SIRT and pooled into two groups, a naïve treatment group and a group presenting PD after immunoembolization [53]. Nevertheless, in this study, most participants of both groups (100% and 91.3%) developed new hepatic lesions while in our study at the moment of progression on SIRT, 50% of the SIRT_IpiNivo group and 21.4% of SIRT group presented progression of extrahepatic metastases while liver metastases were under control. A high number of our patients presented extra liver metastasis at the time of SIRT, whereas patients with extra liver metastases needing treatment were excluded in the study of Gonsalves et al. It is important to note that although the liver is the most frequent site of metastatic disease in mUM, around 50% will also develop extrahepatic disease [2,28]. It is noteworthy to mention the favorable outcomes recently demonstrated with tebentafusp in a phase 3 trial. The estimated OS at 1 year was 73.2% (95% CI; 66.3–78.9) in the tebentafusp group versus 57.5% (95% CI; 47.0–66.6) in the IC group [17]. OS was superior for patients receiving tebentafusp even when the best response was PD (HR = 0.41, 95% CI; 0.25–0.66) [54]. While tebentafusp is currently the best option for mUM patients, demonstrated in a randomized study, its use is restricted to patients with HLA-A*02:01, found in around 50% of patients with UM [55]. Therefore, investigations to establish optimal treatment sequences for mUM are still needed.

The limitations of our study include a small cohort size, a retrospective analysis, and a non-randomized setting. In addition, the baseline characteristics were heterogenous and the order of treatments differed among patients in the SIRT_IpiNivo group. Furthermore, half of the patients in the SIRT group received single-agent immunotherapy, two patients in the SIRT_IpiNivo group had systemic therapy before the immunotherapy and four benefited from liver directed therapy prior to SIRT. Additionally, all patients of this study presented with PD on the first treatment, either SIRT or combined immunotherapy, before receiving the subsequent treatment. Despite these limitations, this is the first report, to our knowledge, comparing combined checkpoint inhibition sequentially with SIRT versus SIRT without ipilimumab plus nivolumab in mUM patients.

## 5. Conclusions

We conclude that combined immunotherapy before or after the SIRT is a safe therapeutic option and appears to be associated with improved survival rates. There were no statistical differences between patients that received upfront immunotherapy compared with patients that received SIRT as first treatment. The approach needs to be further investigated in prospective studies, in particular to define the best sequence of therapies. The hypotheses based on our real-world data might eventually be confirmed with the results of the ongoing NCT02913417 study that is currently evaluating the safety and tolerability of radioembolization and immunotherapy by ipilimumab plus nivolumab treatment started 3–5 weeks after SIRT. 

## Figures and Tables

**Table 1:** Summary of clinical characteristics, treatment features and response to SIRT.

Characteristics	Cohort 1 = SIRT_IpiNivo (*n =* 18)	Cohort 2 = SIRT (*n* = 14)	Total/*p*-Value (*n =* 32)
sex—no (%)			
male	6 (33.3)	7 (50.0)	13 (40.6)/0.55
female	12 (66.7)	7 (50.0)	19 (59.4)
age at diagnosis of primary tumor—no (%)			
median	58	57	58/0.59
range	25–69	31–82	25–82
origin primary tumor—no (%)			
choroid	14 (77.8)	13 (92.9)	27 (84.4)/0.39
ciliary body	2 (11.1)	1 (7.1)	3 (9.4)
unknown	2 (11.1)	0 (0)	2 (6.3)
stage at diagnosis—no (%)			
localized	16 (88.9)	14 (100)	30 (93.8)/0.58
metastatic	2 (11.1)	0 (0)	2 (6.3)
treatment for primary tumor—no (%)			
enucleation	4 (22.2)	3 (21.4)	7 (21.9)/0.58
proton therapy	13 (72.2)	11 (78.6)	24 (75.0)
partial resection	1 (5.6)	0 (0.0)	1 (3.1)
time from diagnosis of primary tumor to metastasis (months)			
median	28.8	21.0	25.1/0.90
range	0–115.7	2.1–153.1	0–153.1
site of metastasis at diagnosis of metastatic disease—no (%)			
liver only	13 (72.2)	10 (71.4)	23 (71.9)/0.64
liver and extra liver	4 (22.2)	4 (28.6)	8 (25.0)
extra liver only	1 (5.6)	0 (0.0)	1 (3.1)
lactate dehydrogenase at 1st SIRT—no (%)			
≤ULN	6 (33.3)	5 (35.7)	19 (59.4)/0.97
>ULN	11 (61.1)	8 (57.1)	1 (3.1)
unknown	1 (5.6)	1 (7.1)	12 (37.5)
liver function tests at start of SIRT—no (%)			
≤ULN	8 (44.4)	8 (57.1)	16 (50.0)/0.72
>ULN	10 (55.6)	6 (42.8)	16 (50.0)
GNAQ and GNA11 status—no (%)			
GNAQ mutation	6 (33.3)	3 (21.4)	9 (28.1)
GNA 11 mutation	8 (44.4)	1 (7.1)	9 (28.1)
GNA mutation (non-specified)	1 (5.6)	0 (0.0)	1 (3.1)
no GNA mutation	1 (5.6)	0 (0.0)	1 (3.1)
unknown	2 (11.1)	10 (71.4)	12 (37.5)
BAP1 status by immunohistochemistry—no (%)			
loss of BAP1	8 (44.4)	1 (7.1)	9 (28.1)
conserved BAP1	1 (5.6)	2 (14.3)	3 (9.4)
unknown	9 (50.0)	11 (78.6)	20 (62.5)
lobes treated by SIRT—no (%)			
only right lobe	7 (38.9)	5 (35.7)	12 (37.5)
only left lobe	1 (5.6)	1 (7.1)	1 (3.1)
whole in multiple sessions	10 (55.6)	4 (28.6)	14 (43.8)
whole liver in one session	0 (0)	4 (28.6)	4 (12.5)
liver directed therapy prior SIRT—no (%)			
none	14 (77.8)	14 (100)	28 (87.5)
surgery	1 (5.6)	0 (0.0)	1 (3.1)
thermal ablation	3 (16.7)	0 (0.0)	3 (9.4)
number of SIRT per patient—no (%)			
1	6 (33.3)	10 (71.4)	16 (50.0)/0.12
2	11 (61.1)	2 (14.3)	13 (40.6)
3	1 (5.6)	1 (7.1)	2 (6.3)
4	0 (0.0)	1 (7.1)	1 (3.1)
if more than 1 session of SIRT, time between first and last SIRT			
1–2 months	9 (50.0)	2 (14.3)	11 (34.4)
3–4 months	2 (11.1)	2 (14.3)	4 (12.5)
5–6 months	1 (5.6)	0 (0.0)	0 (0.0)
presence of extra liver metastasis at SIRT—no (%)			
yes	8 (44.4)	3 (21.4)	11 (34.4)/0.32
no	10 (55.6)	11 (78.6)	21 (65.6)
liver tumor volume (cm^3^)	(*n =* 17)	(*n =* 13)	
median	195.0	165.0	185.0/0.90
range	80–1492	10–1182	10–1492
largest liver metastasis at SIRT			
cM1a (<3.1 cm)	6 (33.3)	7 (50.0)	13 (40.6)/0.47
cM1b (3.1–8.0 cm)	9 (50.0)	4 (28.6)	13 (40.6)
cM1c (8.1 cm or more)	3 (16.7)	3 (21.4)	6 (18.8)
number of liver metastases at SIRT—no (%)			
0–10	7 (38.9)	7 (50.0)	14 (43.8)/0.56
11 or more	11 (61.1)	7 (50.0)	18 (56.2)
dose total received per patient (GBq)			
median	2.4	2.3	2.3/0.66
range	1.1–9	1.3–5.4	1.1–5.4

**Table 2:** Characteristics of treatment and responses to ipilimumab and nivolumab combination.

SIRT_IpiNivo (*n =* 18)	
sequence	
SIRT prior combined immunotherapy	12 (66.7)
combined immunotherapy prior SIRT	6 (33.3)
time between SIRT and combined immunotherapy—no (%)	
median (range)	8.0 (1–31)
1–6 months	9 (50.0)
7–12 months	5 (27.8)
>12 months	4 (22.2)
cycles of combined immunotherapy—no (%)	
1	3 (16.7)
2	3 (16.7)
3	1 (5.6)
4	11 (61.1)
reason for discontinuation of combined immunotherapy—no (%)	
progressive disease	3 (16.7)
toxicity	7 (38.9)
nivolumab maintenance	
yes	11 (61.1)
no	7 (38.9)
number of cycles of nivolumab	
median	13
range	1–33
reason for discontinuation of nivolumab—no (%)	
progressive disease	7 (38.9)
toxicity	4 (22.2)
sites of metastasis at 1st cycle of combined immunotherapy	
liver only	5 (27.8)
liver and extra liver	12 (66.7)
extra liver only	1 (5.6)
Eastern Cooperative Oncology Group (ECOG) performance status at start of combined immunotherapy	
0	12 (66.7)
1	2 (11.1)
unknown	4 (22.2)
lactate dehydrogenase at 1st cycle—no (%)	
≤upper limit of normal, ULN	8 (44.4)
>ULN	9 (50.0)
unknown	1 (5.6)
liver function tests—no (%)	
≤ULN	3 (16.7)
>ULN	12 (66.7)
unknown	3 (16.7)
number of prior systemic treatments—no (%)	
0	16 (88.9)
1	2 (11.1)
type of prior systemic treatments	
chemotherapy	0 (0)
targeted therapy	2 (11.1)
immunotherapy	0 (0)
systemic treatments post combined immunotherapy	
0	9 (50.0)
1	6 (33.3)
2	2 (11.1)
3	1 (5.6)
best overall response to immunotherapy	
complete response	2 (11.1)
partial response	2 (11.1)
stable disease	3 (16.7)
progressive disease	11 (61.1)
distribution of metastases at start of double immunotherapy	
liver	17 (94.4)
lung	10 (55.6)
subcutaneous	7 (38.9)
bone	7 (38.9)
lymph node	7 (38.9)
peritoneum	2 (11.1)
pancreas	1 (5.6)
number of metastatic organ sites at start of double immunotherapy	
1	5 (27.8)
2	2 (11.1)
3	5 (27.8)
4	3 (16.7)
5	2 (11.1)
6	1 (5.6)

**Table 3:** Summary of complications.

Immune-Related Toxicity SIRT_IpiNivo (*n =* 18) *					
Complications	G1	G2	G3	G4	Total
diarrhea/colitis	0	0	4	0	4
hepatitis	0	1	2	2	5
adrenal insufficiency	0	2	0	0	2
thyroiditis	0	4	0	0	4
pneumonitis	0	1	0	0	1
skin rash	0	0	1	0	1
retinal complication	0	1	0	0	1
**Clinical Complications Related to SIRT**					
**Complications**	**G1**	**G2**	**G3**	**G4**	**Total**
SIRT_IpiNivo (*n =* 18)					
abdominal pain	6 (38.9)	1 (5.6)	0 (0.0)	0 (0.0)	7 (38.9)
arterial injury	1 (5.6)	0 (0.0)	1 (5.6)	0 (0.0)	2 (11.1)
bile duct stenosis	0 (0.0)	1 (5.6)	0 (0.0)	0 (0.0)	1 (5.6)
nauseas/vomiting	1 (5.6)	0 (0.0)	0 (0.0)	0 (0.0)	1 (5.6)
gastric ulcer	0 (0.0)	1 (5.6)	0 (0.0)	0 (0.0)	1 (5.6)
abnormal liver tests	9 (50.0)	4 (22.2)	4 (22.2)	0 (0.0)	17 (94.4)
SIRT (*n =* 14)					
abdominal pain	2 (14.3)	0 (0.0)	0 (0.0)	0 (0.0)	2 (14.3)
nauseas/vomiting	1 (7.1)	0 (0.0)	0 (0.0)	0 (0.0)	1 (7.1)
gastric ulcer	0 (0.0)	1 (7.1)	0 (0.0)	0 (0.0)	1 (7.1)
enterocolitis	0 (0.0)	0 (0.0)	1 (7.1)	0 (0.0)	1 (7.1)
abnormal liver tests	8 (57.1)	2 (14.3)	3 (16.7)	0 (0.0)	13 (92.8)

* 12 patients (66.6%) developed irAEs, 7 patients developed one irAEs, 4 patients presented two irAEs and 1 patient had three irAEs.

## Data Availability

All data relevant to the study are included in the article or uploaded as online Supplementary Information (Table S3).

## Supplementary Materials

The following supporting information can be downloaded at: https://www.mdpi.com/article/10.3390/cancers14051162/s1, Table S1: Best liver response to SIRT. Table S2: Univariate analysis of overall survival and hepatic progression-free survival. Table S3: Comparative table of outcomes of different studies using ipilimumab and nivolumab and/or SIRT as treatment for metastatic uveal melanoma. Figure S1: Survival curves and hepatic progression-free survival from SIRT.
