# Supplementary material for: Safety and Efficacy of Ipilimumab plus Nivolumab and Sequential Selective Internal Radiation Therapy in Hepatic and Extrahepatic Metastatic Uveal Melanoma
Source: Cancers (Basel). 2022 Feb 24;14(5):1162. doi: 10.3390/cancers14051162 (PMC8909598; doi:10.3390/cancers14051162)
Supplement: Supplementary file 1 [file cancers-14-01162-s001.zip › cancers-1571292-supplementary.pdf]

cancers MDPI

Safety and Efficacy of Ipilimumab plus Nivolumab and Sequential Selective Internal Radiation Therapy in Hepatic and Extrahepatic Metastatic Uveal Melanoma

Veronica Aedo-Lopez, Camille L. Gerard, Sarah Boughdad, Bianca Gautron Moura, Gregoire Berthod, Antonia Digklia, Krisztian Homicsko, Niklaus Schaefer, Rafael Duran, Michel A. Cuendet and Olivier Michielin

Table S1. Best liver response to SIRT.

Liver response to SIRT at 3 and 6 months				
	SIRT_IpiNivo (n=18)		SIRT (n=14)	
	3 months	6 months	3 months	6 months
Complete Response	2 (11.1)	5 (27.8)	2 (14.3)	2 (14.3)
Partial Response	6 (33.3)	3 (16.7)	2 (14.3)	3 (21.4)
Stable Disease	5 (27.8)	2 (11.1)	6 (42.8)	5 (35.7)
Progressive Disease	5 (27.8)	4 (22.2)	2 (14.3)	1 (7.1)
Not assessed	0 (0.0)	2* (11.1)	2 (14.3)	3** (21.4)
Liver Disease Control Rate	72.2%	55.6%	71.4%	71.4%
Liver Response Rate	44.4%	44.4%	28.6%	35.7%

*one patient died before assessment and one patient was not evaluated due to end of the study. **death or end of life at the time of planned assessment.

Table S2. Univariate analysis of overall survival and hepatic progression-free survival.

Hepatic progression-free survival: univariate analysis			
	Hazard Ratio	95% CI	p-value (log rank test)
Age at diagnosis of metastases (median= 60.5 years old)	0.69	0.32 - 1.48	0.34
Sex (male versus female)	1.32	0.6 – 2.91	0.49
Local therapy for primary tumour (enucleation versus proton therapy)	0.94	0.31 – 2.84	0.90
Presence of extra-liver metastatic disease at SIRT	1.8	0.81 – 3.99	0.14
Treated tumour volume (median= 185cc3)	2.62	1.17 – 5.86	0.016
Treated liver (lobar versus whole)	1.04	0.48 – 2.29	0.91
Number of SIRT (1 versus >1)	1.34	0.63 – 2.89	0.46
Summation of activity by patient (median= 2.3 GBq)	1.20	0.56 – 2.58	0.63
Mean activity by patient (median= 0.6 GBq)	1.33	0.61 – 2.93	0.47
Largest liver metastasis at the SIRT (median= 3.6cm)	1.39	0.64 – 2.36	0.41
Largest liver metastasis at SIRT (0-8 cm versus >8 cm)	3.81	1.29 – 11.25	0.0096
M1a (0-3 cm) versus M1b (3.1-8.0 cm)	1.39	0.60 - 3.26	0.45
M1a (0-3 cm) versus M1c (>8 cm)	0.33	0.10 – 1.09	0.052
M1b (3.1-8.0 cm) versus M1c (>8 cm)	0.23	0.06 – 0.86	0.018
Number of liver metastasis: 0-10, versus 11 or more	0.86	0.39 – 1.90	0.7
Lactate dehydrogenase at SIRT (normal versus >ULN)	1.12	0.50 – 2.50	0.79
Liver function test at SIRT (normal versus >ULN)	1.77	0.80 – 3.88	0.15
**Overall survival from SIRT: univariate analysis**			
	Hazard Ratio	95% CI	p-value (log rank test)
Age at diagnosis of metastases (median= 60.5 years old)	0.66	0.27 – 1.63	0.36
Sex (male versus female)	1.33	0.54 – 3.31	0.54
Local therapy for primary tumour (enucleation versus proton therapy)	1.44	0.33 – 6.42	0.63
Presence of extra-liver metastatic disease at SIRT	2.82	1.04 – 7.66	0.034
Treated tumour volume (median= 185cc3)	2.82	0.98 – 8.17	0.046
Treated liver (lobar versus whole)	0.90	0.36 – 2.27	0.82
Number of SIRT (1 versus >1)	2.13	0.74 – 6.14	0.15
Summation of activity by patient (median= 2.3 GBq)	0.92	0.37 – 2.27	0.86
Mean activity by patient (median= 0.6 GBq)	1.08	0.43 – 2.68	0.87

Largest liver metastasis at SIRT (median= 3.6cm)	2.95	1.05 – 8.30	0.033
Largest liver metastasis at SIRT (0-8 cm versus >8 cm)	6.75	1.95 – 23.35	0.00059
M1a (0-3 cm) versus M1b (3.1-8.0 cm)	1.56	0.55 - 4.42	0.40
M1a (0-3 cm) versus M1c (>8 cm)	0.15	0.04 - 0.63	0.0036
M1b (3.1-8.0 cm) versus M1c (>8 cm)	0.17	0.04 - 0.71	0.0068
Number of liver metastasis: 0-10, versus 11 or more	0.88	0.36 - 2.16	0.78
Lactate dehydrogenase at SIRT (normal versus >ULN)	1.30	0.47 - 3.58	0.61
Liver function test at SIRT (normal versus >ULN)	1.58	0.64 - 3.93	0.32
**Overall survival from diagnosis of metastases: univariate analysis**			
	Hazard Ratio	95% CI	p-value (log rank test)
Age at diagnosis of metastases (median= 60.5 years old)	0.63	0.25 - 1.58	0.32
Sex (male versus female)	1.55	0.62 - 3.88	0.35
Local therapy for primary tumour (enucleation versus proton therapy)	2.41	0.55 - 10.6	0.23
Presence of extra-liver metastatic disease at SIRT	1.55	0.6 - 3.98	0.36
Treated tumour volume (median= 185cc3)	1.97	0.75 - 5.18	0.17
Treated liver (lobar versus whole)	0.73	0.29 - 1.86	0.51
Number of SIRT (1 versus >1)	3.10	1.09 - 8.77	0.026
Summation of activity by patient (median= 2.3 GBq)	0.85	0.35 - 2.07	0.72
Mean activity by patient (median= 0.6 GBq)	0.90	0.37 - 2.21	0.82
Largest liver metastasis at the SIRT (median= 3.6cm)	1.75	0.68 - 4.49	0.24
Largest liver metastasis at SIRT (0-8 cm versus >8 cm)	2.33	0.71 – 7.65	0.15
M1a (0-3 cm) versus M1b (3.1-8.0 cm)	1.43	0.5 - 4.05	0.50
M1a (0-3 cm) versus M1c (>8 cm)	0.35	0.08 - 1.44	0.13
M1b (3.1-8.0 cm) versus M1c (>8 cm)	0.44	0.12 - 1.66	0.21
Number of liver metastasis: 0-10, versus 11 or more	1.26	0.52 - 3.05	0.62
Lactate dehydrogenase at SIRT (normal versus >ULN)	1.23	0.45 - 3.39	0.68
Liver function test at SIRT (normal versus >ULN)	1.27	0.47 - 2.96	0.74

Table S3. Comparative table of outcomes of different studies using ipilimumab and nivolumab and/or SIRT as treatment for metastatic uveal melanoma.

Study	Description of the study/Regimen	Number of patients	ORR to immunotherapy	mPFS (months)	mOS (months)
Zheng 2018	Retrospective. SIRT and any immunotherapy	11	Not available	Not available	17.0 (95%CI: 1.8- 32.2)
Ruohoniemi 2020	Retrospective. SIRT and any immunotherapy	12	Not available for mUM patients	7.8 (96%CI: 3.1-12.4)	17.0 (95%CI: 14.2- 19.8)
Levey 2020	Retrospective. Arm1: SIRT and any immunotherapy within 3m. Arm2: SIRT.	24	Not available	5.2 (95%CI not available)	Arm1: 26.0 (95%CI: 8.9-67.9).<br>Arm2: 9.5 (95%CI: 5.5-19.7)
Najjar 2020	Retrospective. Ipilimumab plus nivolumab.	89	11.60%	2.7 (95%CI: 2.6- 3.3)	15.0 (95%CI: 10.9-21.6)
Piulats et al 2021	Phase II. Ipilimumab plus nivolumab first line.	52	11.50%	3.0 (95%CI: 2.0-4.1)	12.7 (95%CI: 7.1-18.3)
Pelster et al 2021	Phase II. Ipilimumab plus nivolumab any line.	35	18%	5.5 (95%CI: 3.4-9.5)	19.1 (95%CI: 9.6-NR)
Blomen 2021	Retrospective. Arm1: Any immunotherapy plus directed liver therapy. Arm2: other treatments.	42	Not available	Not available	Arm1: 22.5 (95%CI: 15.6-29.3)<br>Arm2: 11.4 (95%CI: 6.3-16.5)
This study	Retrospective. Arm1: Ipilimumab plus nivolumab and SIRT. Arm2: SIRT.	32	22.20%	3.4 (95%CI: 2.6-9.0) for ipilimumab plus nivolumab	Arm1: 49.6 (95%CI: 24.1-NA)<br>Arm2: 13.6 (95%CI: 11.5-NA)

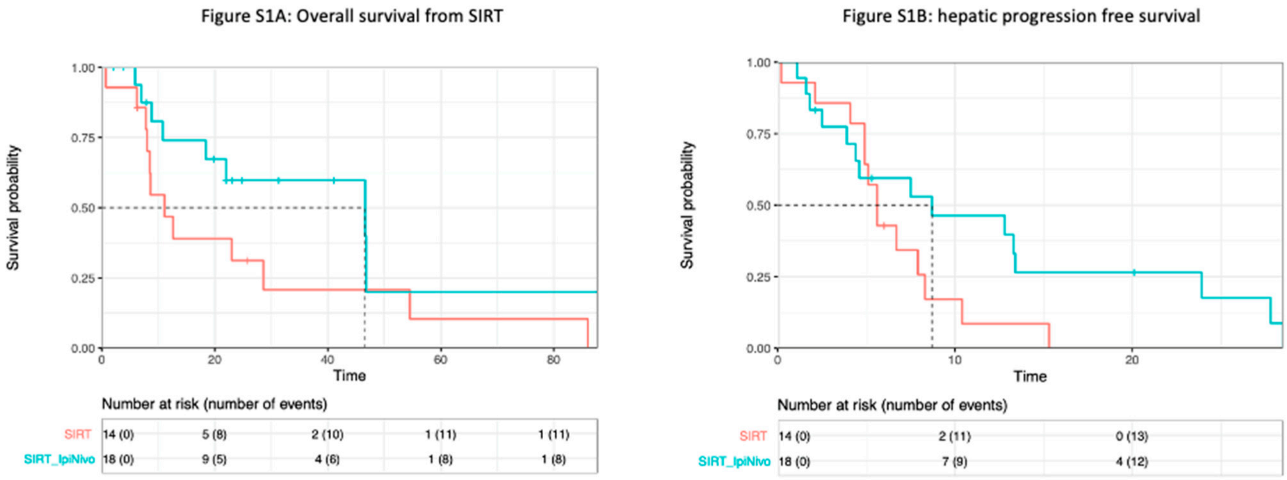

Figure S1. Survival curves and hepatic progression-free survival from SIRT. (A). Overall survival from SIRT. (B). Hepatic progression free survival.
